# GIS-Based Multi-Criteria Analysis Method for Assessment of Lake Ecosystems Degradation—Case Study in Romania

**DOI:** 10.3390/ijerph18115915

**Published:** 2021-05-31

**Authors:** Sorin Avram, Corina Cipu, Ana-Maria Corpade, Carmen Adriana Gheorghe, Nicolae Manta, Mihaita-Iulian Niculae, Ionuţ Silviu Pascu, Róbert Eugen Szép, Steliana Rodino

**Affiliations:** 1 National Institute for Economic Research “Costin C. Kiriţescu” (INCE), Romanian Academy, 13 September Street, No. 13, 050711 Bucharest, Romania; avram.sorin@ucv.ro (S.A.); corina.cipu@upb.ro (C.C.); ana.corpade@ubbcluj.ro (A.-M.C.); carmen.adriana@ince.ro (C.A.G.); mihaitaiulian.niculae@g.unibuc.ro (M.-I.N.); 2 Department of Geography, University of Craiova, Al. I. Cuza Street, No. 13, 200585 Craiova, Romania; 3 Faculty of Geography, Babes-Bolyai University, Clinicilor Street, No 5-7, 400006 Cluj-Napoca, Romania; 4 Department of Biodiversity, Ministry of Environment, Waters and Forests, Libertăţii Boulevard, No. 12, 040129 Bucharest, Romania; nicumanta79@gmail.com; 5 Centre for Environmental Research and Impact Studies, University of Bucharest, Nicolae Balcescu Boulevard No. 1, 010041 Bucharest, Romania; 6 Department of Forest Monitoring, National Institute for Research and Development in Forestry “Marin Drăcea”, 128 Eroilor Blvd., 077190 Voluntari, Romania; 7 Department of Bioengineering, Faculty of Economics, Socio—Human Science and Engineering, Sapientia Hungarian University of Transylvania, Piaţa Libertăţii 1, 530104 Miercurea Ciuc, Romania; szeprobert@uni.sapientia.ro; 8 Doctoral School of Chemistry, Faculty of Natural Sciences, University of Pécs, Ifjúság 6, 7624 Pécs, Hungary; 9 Institute for Research and Development for Hunting and Mountain Resources, Progresului 35B, 530240 Miercurea Ciuc, Romania; 10 National Institute of Research and Development for Biological Sciences, Spl. Independentei, nr 296, 060031 Bucharest, Romania; 11 Institute of Research for Agriculture Economy and Rural Development, Bd. Marasti, nr 61, 011464 Bucharest, Romania

**Keywords:** degradation state, GIS, lake ecosystem, multi-criteria analysis, Romania

## Abstract

In general, the elaboration of the synthesis of water quality in Romania is based on the processing of a large volume of information coming from primary analytical data collected with a constant frequency by the organisms with a specific role in water quality monitoring. This study proposes a novel methodology for multi-criteria analysis aiming to evaluate the degradation state of lake ecosystems. The cornerstone of the newly presented methodology is a geographic information system (GIS) automated tool, involving the assessment of potential degradation sources affecting the watershed that supply the lakes with water. The methodology was tested by performing an analysis on 30 lakes in Romania. The lakes belong to different geographical areas, owing various natural specific conditions and were selected to fit to various types and specific local conditions. The calculation of the WRASTIC-HI (Wastewater–Recreation–Agriculture–Size–Transportation–Industry–Cover–Hazard Index) revealed that, out of 30 lake ecosystems selected as the case study, two lakes were fully degraded, 24 lakes were semi-degraded, and four were in a natural state. The four lakes characterised by a natural state are located in mountainous regions or in the Danube Delta. The results obtained on the selected lakes proved that the proposed index calculation corresponded in all case studies to the real field situation, highlighting thus the accuracy of the assessing process and increased advantages of the assessment’s automation.

## 1. Introduction

Terrestrial and aquatic ecosystems offer a series of services which contribute to human well-being [1], defined as a series of benefits obtained from these natural ecosystems [2,3] and refer to provisioning services, regulating services, cultural services and supporting services [2]. However, ecosystems are permanently subjected to degradation, as a result of diversification of anthropic activities for the purpose of satisfying the human needs, including by use of resources offered [4]. Recently, a non-sustainable use of ecosystemic services at world level has been recorded [5,6]. The degradation of natural habitats, including aquatic ecosystems, represents the most important cause of loss of biodiversity [6]. The main indirect causes of degradation of aquatic ecosystems, including lake ecosystems are represented by the increase of population and economic development [7], with direct effects on species and reduction of populations of species. These two drivers are directly linked to the development of infrastructure, change of land cover, overexploitation and the introduction of invasive allogeneic species [2,8,9], drainage and irrigation systems, and chemical pollutants [10,11,12]. The aquatic ecosystems, including lakes ecosystems, are also affected by climate change, the seasonal thermal stratification of lakes being modified over time [13,14].

Preoccupations of decision-makers regarding evaluation of degradation and reconstruction of water bodies’ conditions materialized in the elaboration of strategies and policies in the field at a global scale [15]. The European Union (EU) Water Framework Directive establishes a common framework for implementation of actions at community level in the field of water-related policies It establishes a common framework for the sustainable and integrated management of all water bodies (groundwater, inland surface waters, transitional waters and coastal waters) and requires that all impact factors as well as economic implications be taken into account [16,17]. In Romania, provisions of this directive were transposed into national legislation by the no. 107 Law of 1996, amended and updated in 2015 by the no. 196 Law [18].

Later on, in 2013, in order to evaluate the changes in the ecosystems, the EU created a strategic framework through the VIIth Program of Action for Environment [19]. At EU level, according to the results of evaluation carried out in 2015, conservation status of habitats and species was still not adequate requiring significant efforts to achieve targets set in EU Biodiversity Strategy to 2020 [20]. The protection and preservation of species and habitats of community importance represented the main objective of the European Union with regard to biodiversity preservation strategy [21]. This strategy aimed at maintenance and reconstruction of ecosystems and their services and reducing the loss of natural habitats, including rivers and lake ecosystems. The European Strategy for Biodiversity (2011–2020) took over the Aichi targets for biodiversity set in the Convention for Biological Diversity [22].

The EU’s new biodiversity strategy for 2030 was released in May 2020. This strategy and its associated action plan represent a comprehensive, ambitious, long-term plan to protect nature and reverse ecosystem degradation [23]. This strategy makes reference to the Water Framework Directive objective to reach a good ecological status by 2027 for all EU rivers, lakes, transitional and coastal waters including wetlands.

Therefore, to be able to translate into practice the objectives of the latest EU action plans towards transition to a more sustainable society (including the Biodiversity Strategy released in 2020) it is necessary to evaluate the changes induced by humans in ecosystems. This will help in understanding where the transformations were caused and at what scale [24], and to properly design the actions that should be implemented for the reduction of negative effects.

The purpose of this study is to elaborate a methodology by which the evaluation of state of degradation for lake ecosystems from Romania can be undertaken using geographic information system (GIS) techniques, a methodology that can be further used at an even wider scale, regardless of the type and territorial localization of lakes. This study was aimed at (i) creating an automated GIS specific tool to determine degree of degradation of lake ecosystems and, furthermore, their integration in one of the degradation classes (degraded, semi-degraded and natural), and (ii) testing and validating proposed methodology on relevant case studies selected from Romania.

## 2. Materials and Methods

### 2.1. Type and Source of Primary Data Used

The data structure design and the processing methodology is presented in Supplementary Table S1.

Mapping of the lake ecosystems was done in accordance with the classification system of habitats European Nature Information System (EUNIS) level 2, in which lake ecosystem is considered to be a water surface area [25] formed by two major components: pelagic area and seaside area. Riparian zone was not included in delimitation process, although it has certain important structures and functions [26,27], but some characteristics of riparian ecosystems were used for assessing the state of lake ecosystems. Type and source of used data as well as the intermediary data processing, are listed in Table 1.

### 2.2. Indicators Used to Assess the Level of Degradation for Lake Ecosystems

For an appropriate analysis of degradation, seven indicators were used for the calculation of the WRASTIC (Wastewater–Recreation–Agriculture–Size–Transportation–Industry–Cover) index and 3 indicators for the calculation of the Hazard Index (HI). The used indicators and sub-indicators are listed in Table 2 and Table 3. The selection of indicators was made in relation to their potential in affecting the state of lake ecosystems and to availability of data at national level.

### 2.3. Calculation of the Indicators to Assess the Degradation State of Lake Ecosystems

The proposed methodology to calculate selected indicators is derived from three indexes known and validated by peer-reviewed literature in the field. These indexes can be applied regardless of geographical context, starting from the characteristics of watersheds and degradation sources identified in the area surrounding the water bodies. These indexes are: PPL (potential pollutant load) [28], WRASTIC [29] and LV (lake vulnerability) [28]. Elements specific to first two indexes were calculated based on information regarding the land use categories that are found in the watershed’s area, considering each type of land use to be associated with a specific degradation potential. In this respect, the combination of the two indexes seeks to illustrate the influence of human activities on lake ecosystems. The combination of morphological data (slope and aspect) and pedological data, as relevant factors in filtration of polluted waters on their route to accumulation point, defines the third index.

**Figure 1:**
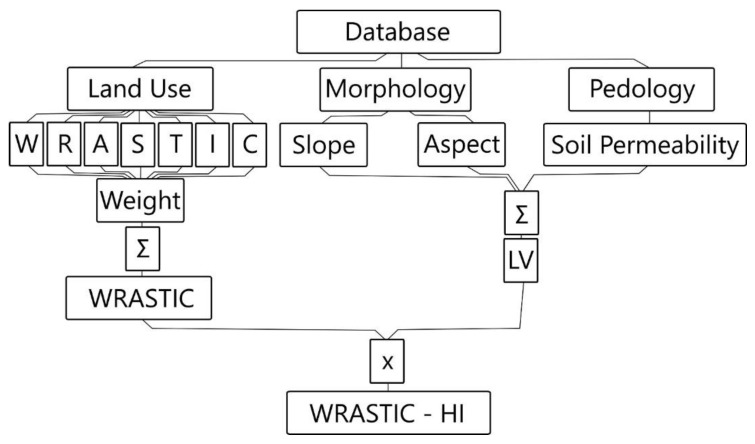
Logical scheme of WRASTIC-HI (Wastewater–Recreation–Agriculture–Size–Transportation–Industry–Cover–Hazard Index).

Aggregation of these three indexes led to development of a new index called WRASTIC-HI (Figure 1), whose applicability implies a weighing procedure of indicators listed in Table 4 and Table 5.

For each of the indicators, the value was calculated as described above. Having the values calculated, these were reclassified in order to be integrated in one of the three quality classes of the ecosystem analyzed: lake ecosystems in natural state, lake ecosystems in semi-degraded state, lake ecosystems in degraded state, with an interpolated extent of their values.

The higher the WRASTIC value, the higher the degradation of ecosystem.

WRASTIC-HI in detail means:

- Wastewater discharge (W) or discharge of wastewaters resulted from anthropic activity on the territory delineated by the watersheds;
- Recreational land use impacts (R) or impact of recreational activities;
- Agricultural land use impacts (A) or impact of agricultural activities;
- Size of watershed (S) or size of watersheds feeding the lake;
- Transportation avenues (T) or influence of the means of transport;
- Industrial land use impacts (I) or impact of industrial activities;
- The amount of vegetative ground Cover (C) or percentage of coverage with vegetation;
- Hazard Index (HI) or risk Index; this includes Permeability (P) or soil permeability, Aspect (E) or aspect of the slopes and Slope (S) or degree of inclination.

The equation for the calculation of WRASTIC-HI is:

WRASTIC-HI = (Wn × Wp + Rn × Rp + An × Ap + Sn × Sp + Tn × Tp + In × Ip + Cn × Cp) × (Sn + En + Pn)(1)

where n is the grade of each analysed factor, p is the weight, W, R, A, S, T, I, C represent the indicators proposed for analysis, described in Table 1 and Table 2.

For the calculation of the aforementioned indexes, ArcGIS software [30] and Python programming language were used. The method proposes automation of the calculation of all factors and generation of an aggregate degradation index.

Using an iterative algorithm developed in Python, a programming language frequently used in geospatial analysis, the main specific functions of ArcGIS can be employed. Their correlation by the method of favorability classes and rasterial calculation ensures a customized result after each data source used.

**Figure 2:**
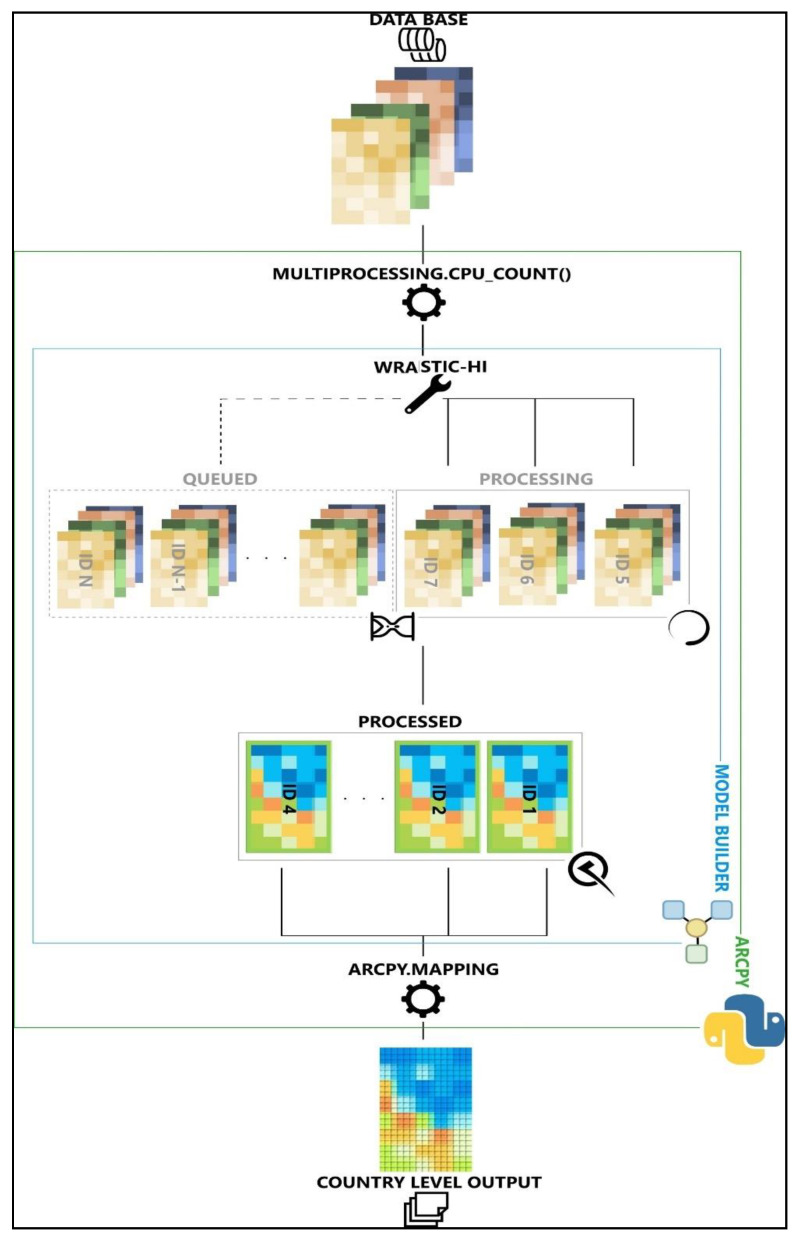
Workflow representation.

As the use of several ArcGIS-specific tools is necessary, we resorted to ModelBuilder as a visual programming environment. It allows for development and change of processes and work flows by intervention on diagrams of geoprocessing tools. The result is an iterative structure of operations, grouped in ArcToolbox-es, similar to any other GIS tool and having a user-friendly interface. The data which will be used by the tool, a collection of vector and raster files described in Table 1, was stored in a geodatabase (.gdb) type database in order to facilitate and optimize utilization taking into account the increased number and variety of the GIS input data. The .gdb reduces data storage requirements and facilitates data transfer and usage from several devices (Figure 2).

Multiple criteria decision-making (MCDM) or multiple criteria decision analysis (MCDA) is a workflow design focused on structuring and solving problems involving multiple criteria when no unique optimal solution is known. This can be seen as choosing either the best alternative from a set of available alternatives or the most efficient nondominated (not improvable in any criterion without sacrificing in another) alternative [31,32].

Hence, the processing approach needed to be a tool capable of incorporating expert judgement weights and thresholds while in the end its role was to help highlight the optimal alternative by tradeoff between certain criteria or data sets and others.

Whether it is an evaluation problem or an analysis one, preference information of DMs is required in order to assess optimal solutions. This is where the advantages of arcPy site package over standard ModelBuilder became a necessity. From trivial aspects, like the fact that ModelBuilder has no mapping capabilities, whereas arcpPy mapping does, or text manipulation, to advantages on the arcPy side regarding workflow optimization such as the multiprocessing package or parallel processing, nested loops and other iterative logic tools, all contributed to optimizing the workflow. Accomplishing a similar result in ModelBuilder would have required constructing intricate nested models, difficult to debug and follow, without any control once run, and without rollback in case of errors or crashes.

As a consequence, the proposed solution is an encapsulated workflow that builds around ModelBuilder’s optimization towards common tool use and its independence from debugging procedures for short, concise workflows. Despite containing arcpy procedures, its integration with ArcGIS software was undertaken as in the case of any other preexisting tool, an aspect essential to migration from the developer’s machine to operator’s ones. Built for reuse, we considered this solution to be a perfect approach for both exploring and testing what-if scenarios in initial supervised stages, as well as for full-on iterative processing of country level data, once mature enough.

The general workflow required pre-processed country-scale datasets (to the level of final classes definition) as input for a multi-stage iterative MCDM approach. This allowed for parallelized processing on all the levels of the WRASTIC-HI approach simultaneously (wastewater, recreational activities, agricultural activities, watershed size, transportation infrastructure, industrial activities, natural vegetation cover, geo-morphology and soil conditions). Each individual branch of the algorithm differed, based on its specificity, but all of them gravitated around the preexisting 3D analyst and analysis tools as well as the more general data management tools for various spatial interaction analysis, conversion, aggregation or reclassification, attribute manipulation and joining as well as data fixing, debugging and validation.

The association between WRASTIC index and HI index is made in such way that it takes into account the local natural morphological conditions. Thus, three types of lake ecosystem have been established, depending on the values of HI:

- type 1—lake ecosystems inclined to a small degree towards the accumulation of pollutants (lower slope values, permeable soil, slopes not directly exposed to the lake);
- type 2—lake ecosystems inclined to an average degree to the accumulation of pollutants (average slope, soil with average permeability, intermediate aspect with regard to the lake);
- type 3—lake ecosystems inclined to a large degree towards the accumulation of pollutants (high slope values, impermeable soil, slopes directly exposed to the lake).

Depending on these categories, the inclusion in degradation classes is as follows:

Lake ecosystems with HI type 1:

- Ecosystems in natural state: WRASTIC values between 0–30;
- Ecosystems in semi-degraded state: WRASTIC values between 31–63;
- Ecosystems in degraded state: WRASTIC values between 64–100.

Lake ecosystems with HI type 2:

- Ecosystems in natural state: WRASTIC values between 0–27;
- Ecosystems in semi-degraded state: WRASTIC values between 28–58;
- Ecosystems in degraded state: WRASTIC values between 59–100.

Lake ecosystems with HI type 3:

- Ecosystems in natural state: WRASTIC values between 0–21;
- Ecosystems in semi-degraded state: WRASTIC values between 22–44;
- Ecosystems in degraded state: WRASTIC values between 45–100.

The final result is defined by zonal statistics > maximum, whilst the percentages of all the classes are recorded. When the work flow is finalized for one of the geometries of the lake layer, the iteration continues with the next polygon. The cycle continues until it completes the entire database and all that is left to be done is the generation of a thematic map with quality classes symbolized based on .lyr files attached to the database.

### 2.4. Validation of the Proposed Methodology

In order to validate the proposed methodology, we have calculated the newly developed index for 30 lakes situated on Romanian territory. These lakes were selected to fit to various types described above and to specific local conditions.

**Figure 3:**
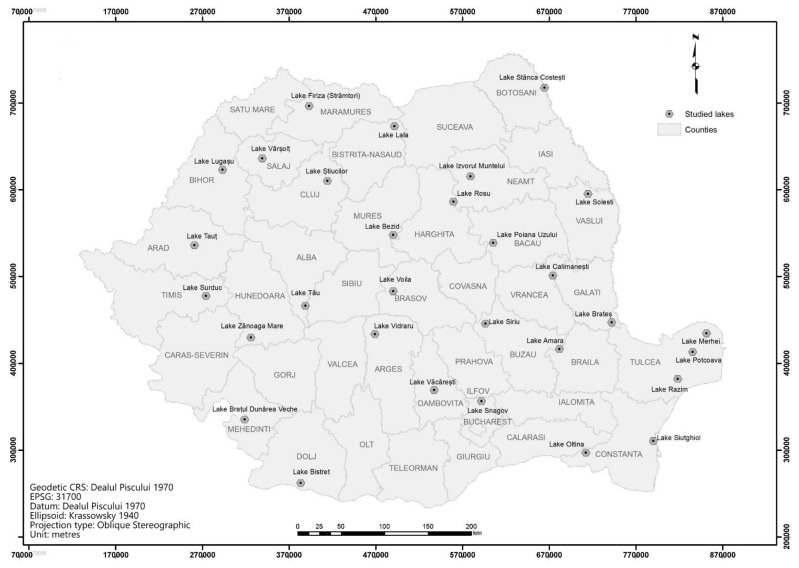
Localization of the assessed lake ecosystems.

The assessed lakes are listed in Table 6 and illustrated in Figure 3. The validation of the results was undertaken based on the cartographic support, topographic maps scale 1: 25,000, and with the aid of orthomosaics and satellite images.

## 3. Results and Discussion

The process of assessing the degree of degradation of lake ecosystems involved several intermediate analyzes, respectively obtaining specific data for calculation of the selected indicators.

**Figure 4:**
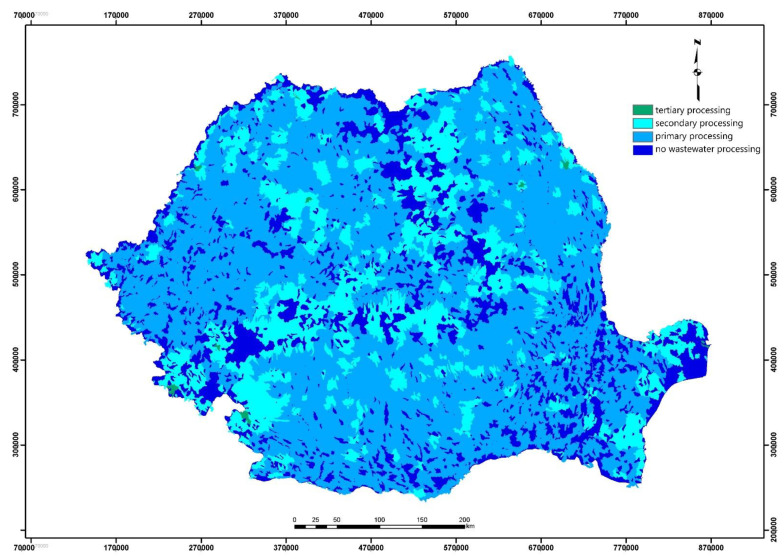
Initial processing of input data: wastewater processing.

For the calculation of wastewater, the two rasterial sets were generated by interpolation, to locate the nearest sampling points and for reclassification for the data of aggregation of the dependent population with the locations of the treatment plants. The data set obtained (Figure 4) was saved for use in the WRASTIC index calculation formula.

**Figure 5:**
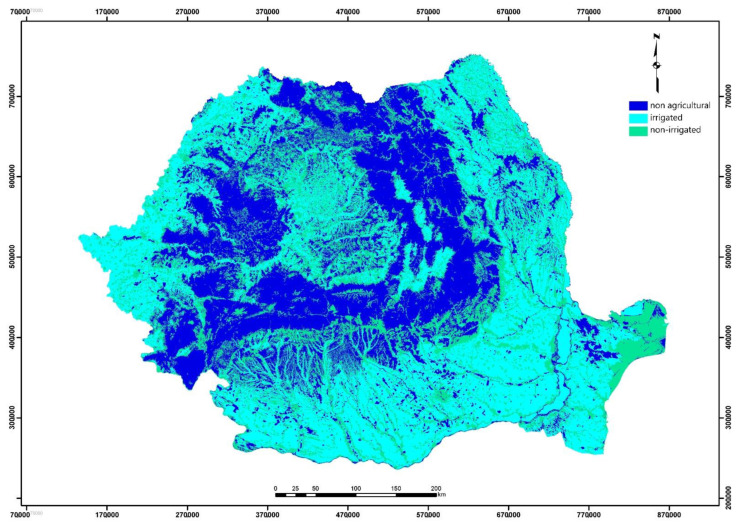
Initial processing of input data: agricultural activities.

These data reflect the influence of human settlements and the ability of wastewater processing plants to cope with the flow of wastewater generated by the existing population in the vicinity of lake ecosystems. The class of agricultural activity identifies the existence of land intended for agriculture in the region of river basins that supply the lake (Figure 5).

**Figure 6:**
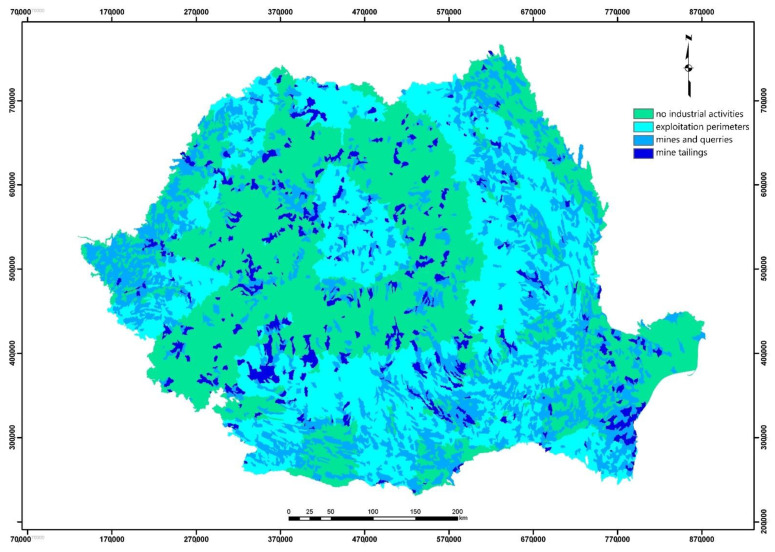
Initial processing of input data: industrial activity.

The influence of industrial activity was similar, but used different data sources. The final correction of the classes was made according to the identification of the position of the lake in a perimeter of exploitation or even in the area of active industrial units with high pollutant potential (Figure 6).

**Figure 7:**
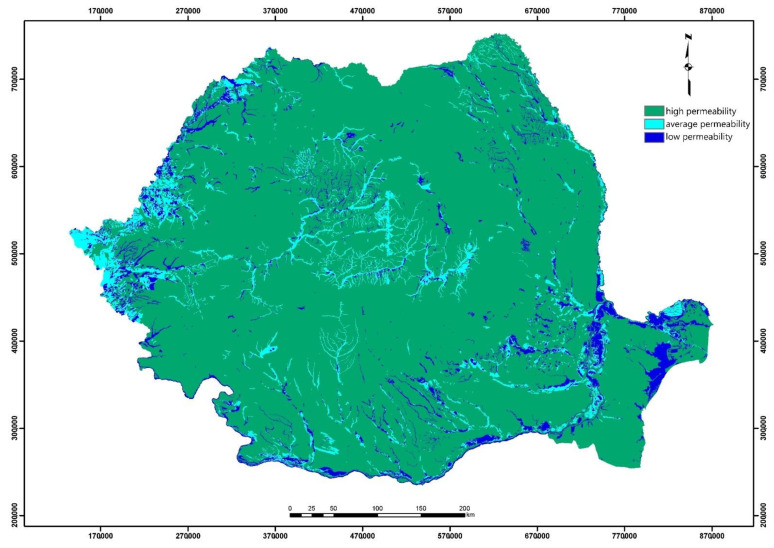
Initial processing of input data: soil permeability map.

For soil permeability, starting from the physical data of both the upper and deep soil layers, favorability classes were generated (Figure 7). Soil permeability was derived from the textural classes associated with the soil types represented in the pedological map of Romania.

The results obtained from the indexes that define the WRASTIC-HI index, in the context of the selected methodology, are relevant and help justify the three general state of degradation identified (Table 7 and Table 8).

As far as Industrial land use impacts (I) is concerned, the value corresponding to a certain lake is influenced by presence of industrial activity or exploitation activities in area surrounding the lake, and also by being considered to be part of the exploitation areas.

Among the 30 lakes studied, 57% of them were evaluated with the value 0, due to lack of industrial influence or exploitation perimeters in their proximity, values highly dependent on selected buffer size (e.g., Vidraru Lake, Surduc Lake, Zanoaga Mare Lake, Rosu Lake, Razim Lake). The vast majority of these lakes are localized in different categories of protected area, especially in mountainous regions.

These values are significantly different than those identified for lakes Vacaresti, Siriu, Bratul Dunarea Veche, Siutghiol etc., which due to their position inside large urban centers (Vacaresti Lake), or near industrial units and being impacted by resources exploitation, their water supply comes from surrounding watersheds where these anthropic activities are carried out.

Recreational land-use impact (R) is calculated considering lake accessibility, tourist infrastructure surrounding the lake (up to 50 m) and regulations regarding motorized sport activities allowed to be performed on lake. In most cases (80%), law permits various motorized vehicles to access the lake area and its surface, which led to higher amendments being applied.

Although Lake Snagov is part of Snagov Nature Reserve, restrictive factors such as county and communal roads leading to lake (DJ111, DC101B, etc.), the presence of tourist infrastructure and motorized vehicles being allowed to hover on the lake surface have resulted in higher amendments. Due to its position near an urban center, same type of amendments has been applied to Lake Calimanesti, although this lake is not in a protected area. The lakes that suffered the lowest correction rates are those from within protected areas that cannot be accessed by car, where there is no tourist infrastructure and no water sports allowed (Lake Potcoava, Lake Lala, Lake Zanoaga Mare).

Regarding wastewater discharge (W) index, focus was on the method of processing wastewaters for Lake Merhei (Danube Delta Biosphere Reserve) and Lake Brates (near the city of Galati), identified as relevant due to their different positions compared to human settlements. Therefore, we were able to analyse the human impact in the area and the poor efficiency of the water processing plants in handling the generated wastewater flow. The lake inside Danube Delta suffers no influence from human settlements or waste water processing, while Lake Brates is highly impacted by the 1st, 2nd and 3rd levels of waste water processing coming from the water treatment plants and is thus subjected to higher amendments.

Size of watershed (S) index highlights the impact that the size of watershed feeding the lakes can have on lakes. It works as a weighting factor for the other indexes, as the level of potential pollutants is also closely linked to area from where they get collected.

Lake Bistret for instance, collects its water from a larger area, with a wider channel network, compared to Lake Poiana Uzului. The latter is positioned in a mountainous area, more fragmented from a morphological perspective. This specific localization restricts the expansion of the watershed that feeds the lake waters [33].

Transportation avenues (T) index analyses the influence various ways of transport have on the lake’s state. It takes into account the entire watershed and is in close relation with the highest category of roads crossing it. The lakes in scope for this study display various values for this index, due to means of transportation in its proximity being in very different stages of development. For lakes in Danube Delta—Lake Merhei and Lake Potcoava—due to having no road or rail infrastructure near it, the (T) values were minimal. The highest values, that also determine a significant amendment, have been identified for Lake Siutghiol. Here, the lake’s watershed is crossed by the European Road E87, A4 Motorway, as well as several other national and local roads. Same situation has been identified for Lake Lugasu, where the European Road E60, county road DJ1081 and the railway linking Oradea to Vadu Crisului cross the surface of the watershed.

The degree of vegetation coverage is underlined by the amount of vegetative ground Cover (C) index. The results of this index have proven to be in line with level of vegetation that is capable of filtering the various pollutants within the watershed.

A number of 14 lakes (47% of total number of analyzed lakes) are characterized by a percentage of coverage with vegetation of over 50%, receiving the lowest grades, materialized by a low rate (Surduc Lake, Vidraru Lake, Siriu Lake etc.). A degree of coverage with vegetation below 5%, equivalent to a high rate, is recorded for the lakes Bistret and Brates, located in regions with almost permanent agricultural activities where forests were missing entirely, due to largely being clear-cut and converted into agricultural fields [34].

Agricultural land use impacts (A) index, analyses the influence of agricultural activities and is again, a composed one, based on different weights, dependent on the irrigated surfaces or the area affected by drought and the number of agricultural activities.

A high number of lakes (70% of the total analyzed number) are characterized by permanent irrigation below 10% (Snagov Lake, Amara Lake etc.), while Bezid Lake is characterized by a high irrigation degree (between 50% and 75%).

The calculated indexes are weighted according to the proposed methodology, being corrected later based on morphological and pedological criteria. The grades obtained previously were corrected if the lake was located in a region with steep slopes, aspects oriented towards the water surface and impermeable pedological substrate.

**Figure 8:**
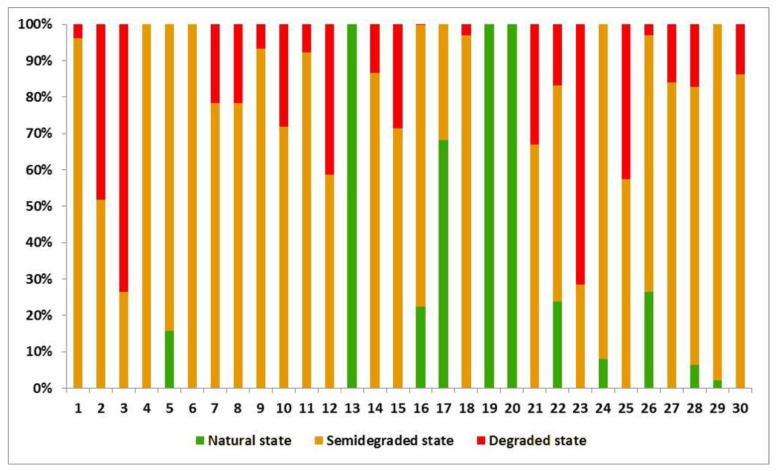
Degradation state of analyzed lakes (%).

**Figure 9:**
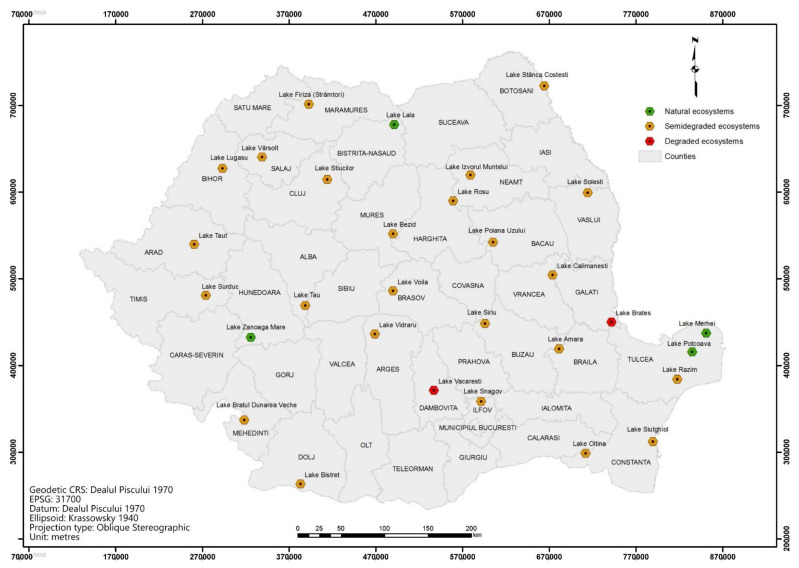
Degradation state of studied lake ecosystems.

After the calculation for each index, the results were summed up in weighted manner, obtaining thus, the degradation levels which affect each lake separately. The dominant class was established statistically as well as the proportion to which the lake is affected by each of them (Figure 8 and Figure 9).

Following the evaluation of the degradation state of lake ecosystems by calculation of WRASTIC- HI index, out of 30 lake ecosystems selected as case study, two lakes are fully degraded (Vacaresti Lake and Brates Lake), 24 lakes are semi-degraded, and four are in a natural state. The four lakes characterised by a natural state are located in mountainous regions as well as in the Danube Delta (Zanoaga Mare Lake, Lala Lake, Potcoava Lake and Merhei Lake).

**Figure 10:**
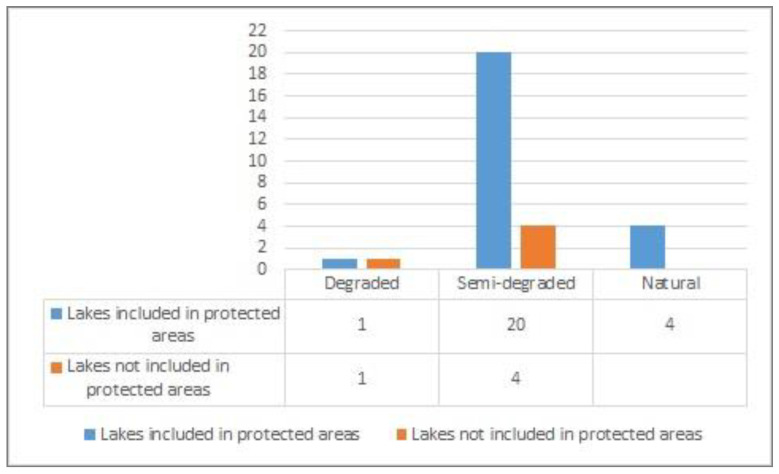
Degradation state in relation to nature protection areas.

The lakes included in nature protected areas are not prevented from human impact, as 20 lakes in this situation were found as semi-degraded, four are in natural state, while Lake Văcăreşti is degraded in spite of its protection regime, due to its location in a large city, namely Bucharest (Figure 10).

**Figure 11:**
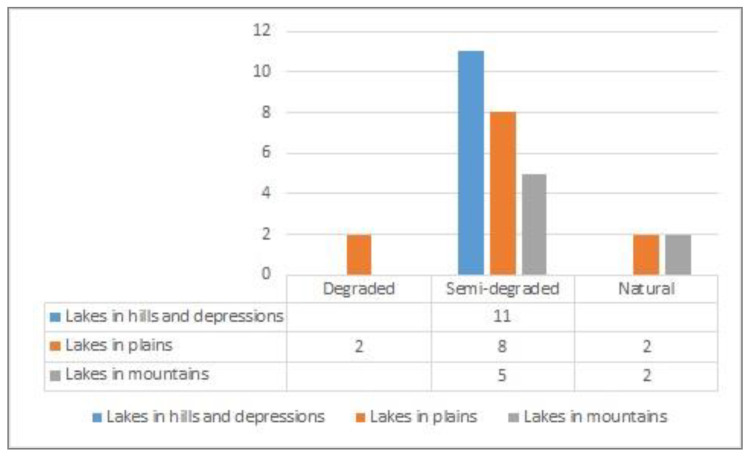
Degradation state in relation to major geographic units.

The lakes located in hills and depressions are all in semi-degraded state, while those in plains fall in all the three categories, with a majority in the semi-degraded category. The lakes in the mountainous regions are generally less-affected by human activities, but at the same time, they might be fragile from the point of view of slope inclination and permeability, which might privilege the accumulation of pollutants (Figure 11).

The use of GIS techniques, remote sensing and aerial imagery for various environmental studies is beginning to gain space in Romanian research as well, ranging from urban development, mining industry, water and forest management, biodiversity conservation, and cultural heritage [35]. GIS techniques were employed for the estimation of the area and depth of a lake in NW Romania after ecological restoration of the wetland. The result was a complex model that is able to develop multiple scenarios [36]. Some other authors had used GIS techniques for determination of various parameters of water ecosystems. For example, Rosca et al. [37], evaluated the landslide hazard in the drainage basin of the Niraj River. Their aim was to include as far as possible the dichotomous relationship between space and time. The authors approached a combined method including GIS techniques for quantitative analysis and statistical analysis and detailed observation in the field, both directly and indirectly through remote sensing.

Indexes used towards establishing degradation state of ecosystems can be classified in several categories, respectively indexes which take into account certain target species of ecosystem, the ratio between classes of organisms, specific chemical compounds, trophic levels, composite indexes, holistic indexes, thermo-dynamic indexes [38]. These indexes cannot be applied to all types of ecosystem, so certain criteria are necessary for their selection.

One of the most commonly used indexes for evaluating the lakes’ state of degradation is the saprobic index. Based on it, the water of lakes or rivers can be classified in: oligosaprobic water (unpolluted), beta-mesosaprobic water (slightly polluted), alpha-mesosaprobic water (polluted) and poly-saprobic water (very polluted). Suggestive in this analysis are also the Nygaard index which highlights the organic pollution of water bodies based on the analysis of ratios between planktonic algae which develop in these water bodies [39].

Research in this field had as result development and calculation of a significant number of indexes by which we can evaluate state of a water body: the benthic response index, which analyses tolerance to pollution of species characteristic of target ecosystem [40]; the conservation index, which aims at the analysis of bio-accumulative species such as mollusks, which can highlight the presence of toxic elements in ecosystem [41]; the Shannon–Wiener index with role in analysis of proportion of individuals within species, the lowest values of it highlighting degradation of the ecosystem [42]; the trophic index, used to highlight the natural degree of the analysed ecosystem [43]; pollution index with role in the analysis of pollution effect on the number of individuals from indicative species of ecosystem [44]; the benthic index, used to determine the make difference between degraded and non-degraded ecosystems based on abundance of bivalves in water [45]. Also, the health index of ichtiofauna is very important, highlighting the ratio between natural ichtiofaunistic potential of a water body and its real potential at the moment of analysis [46].

A more systemic approach applied by Huang et al. [47] by calculating a regional water environmental capacity using a mathematical model. This kind of assessment has the advantage of being able to further use of data for predicting the future state of the examined ecosystem resulting from different management strategies applied to specific parameters [48].

Moreover, multi-criteria analysis represents a versatile tool for combining both qualitative and quantitative data for the assessment of spatial and temporal distribution of environmental issues [36,49].

An example of such multicriteria index used for evaluation of the state of degradation of lake ecosystems is WRASTIC index. This index is analysing the presence of degradation sources in supplying the basin. WRASTIC index analyses pollution risk, respectively degradation sources, from the watershed that feeds a water body [29]. The PPL index also analyses presence and intensity of potential pollution sources from the drainage area of lakes, with the purpose of establishing degradation classes of the water body [28]. The lake vulnerability index highlights the capacity of the water body to handle the impact generated by degradation sources, taking into account parameters like slope, soil permeability or aspect of slopes [28]. These categories of indexes represent scientific support on which development of our proposed methodology relies on. Such activities are necessary for fulfilment of our country’s incumbents with regard to the implementation of strategic documentations that exist at European level.

To the best of our knowledge there is no such study developed for Romania, regarding the development of a multicriteria analysis based on available data, for the assessment of lake ecosystems that could be replicated for the entire country. Most of the indicators that we have applied are WRASTIC specific indicators, used so far in studies in the literature, but this newly developed methodology proposes to modify and supplement them with values and classes taken from the PPL index, such as the degree of nature conservation in the area of the lake ecosystem (Nature Conservation).

In all case studies, meaning 30 lakes, the results obtained by assessing degradation through the calculation of the WRASTIC-HI index coincided with the real conditions in the field.

Although the algorithm calls for multiple functions whose use requires experience (3D analyst, conversion, spatial analyst, data management, multidimensional, coverage, analysis, spatial statistics), reducing them to a minimum of input data eliminates the human component that could bring errors in the final result.

Thus, the advantages of such a system, start from the first step, the acquisition of data from open and validated sources and continue through the whole process: the use of a single software for the entire workflow eliminates the possibility of interconnection errors and component interpretation spatial data; the presence of a user-oriented interface, regardless of its specialization, or experience in the field of remote sensing or GIS; reducing the time and resources required for processing.

WRASTIC-HI index processed through the ArcGIS software allows for the performance of analyses regarding the state of lake ecosystems at national level to be increased, despite the fact that the accuracy of final values is dependent on quality of used data.

## 4. Conclusions

The proposed methodology is based on multi-criteria analysis and GIS techniques and used open-source data which transforms it into a very useful tool when intending to assess ecosystems on large areas, at national or even European level, with reduced time and financial effort, taking into account that field analyses are difficult to be conducted at these levels, even if they could provide studies with increased accuracy. However, like any automated instrument which uses derivative data and work flows, the accuracy of results depends on the accuracy and detail level of the databases used. The methodology could be also applied at local level, in this cases input data could be achieved through field investigations and thus results could be even more improved. As such, the higher the degree of confidence of input based data, the higher the accuracy of results.

The methodology was tested and validated on 30 lake ecosystems in Romania, the results of index calculation corresponded in all case studies to the real field situation, highlighting thus the accuracy of the assessing process and increased advantages of assessment’s automation.

## Figures and Tables

**Table 1:** Data sources and intermediary data processing.

Source	Data (Type)	Description	Use
Copernicus Land Monitoring Service	Corine Land Cover v.2012 (Polygon)	Double coverage of satellite images was used. The mapping was done by computer-assisted photo-interpretation technology.	Calculation of Wastewater–Recreation–Agriculture–Size–Transportation–Industry–Cover (WRASTIC) Index
Copernicus Pan European High-Resolution Data	Permanent Water Bodies v.2012 (Polygon)	Information on the various land use categories, in high resolution. The delimitation of water bodies was done as a binary product (presence/absence). Includes the permanent water bodies delimited with a spatial resolution of 20 m.	Identification of lakes
European Environmental Agency	Digital Elevation Model over Europe (EU-DEM)	EU-DEM with a 25 m resolution and vertical accuracy of +/− 7 m Root Mean Square Error (RMSE), based on Shuttle Radar Topography Mission (SRTM) and Advanced Spaceborne Thermal Emission and Reflection Radiometer (ASTER) Global Digital Elevation Model (GDEM). The original reference system is The European Terrestrial Reference System 1989 (ETRS89). The tiles being aggregated 100 × 100 km tiles re-projected in TRS-LAEA reference system.	Calculation of Hazard Index (HI)
European Environmental Agency	Major sources of pollution (Point)	The major sources of pollution were extracted from the European Pollutant Release and Transfer Register (E-PRTR), which contains reports of over 30,000 facilities with high polluting potential with a coverage of 65 economic activities from EU countries.	Calculation of WRASTIC Index
Open Street Map (OSM)	OSM dataset (Polygon)	The data are obtained by systematic analyses of the land any changes being introduced in the OSM database via a supervised review. The availability of satellite data and photogrammetric images led to an important increase of the automation level.	Calculation of WRASTIC Index
National Agency for Mineral Resources	Exploitation perimeters (Raster)	The map was done following the conclusion of the exploitation agreements and development plans of all-natural resources.	Calculation of WRASTIC Index
Ministry of Environment	Special Areas of Conservation (SAC), Sites of Community Importance (SCI) and Special Protection Areas (SPA) Limits (Polygon)	Delimitation of SAC, SCI and SPA, part of Natura 2000 network.	Calculation of WRASTIC Index
European Soil Data Centre	Two-Sided Geometric Distribution (TSGD) Eurasia (Polygon)	The data was developed for the use of Land Resource Management agencies, of the Joint Research Centre of EC, in collaboration with the European Soil Bureau Network.	HI Index
European Environmental Agency	Urban Waste Water Treatment, Agglomeration—overall compliance (Point)	Information on the implementation of Directive UE 27—Urban Waste Water Treatment: localization of treatment plants, the processing stages of the wastewater and the processing degree compared to scale of production.	WRASTIC Index
National Agency of Cadaster and Land Registration	Administrative Boundary stOrder (Polygon)	Data regarding the structure of the Romanian territory in Local Administrative Units (LAU) units	Mapping of lakes
United States Geological Survey	Landsat 8 (Raster)	Satellite imagery with a spatial resolution from 15 to 100 m, global scale. Landsat 8 operates in visible infrared spectrum, close infrared and thermal infrared spectrum.	Validation of results and control

**Table 2:** Indicators used in the calculation of the WRASTIC (Wastewater–Recreation–Agriculture–Size–Transportation–Industry–Cover) index.

No	Indicator	Sub-Indicator
1	Wastewaters (W)	Aggregation nuclei
		Treatment plants
2	Recreational activities (R)	Aquatic sports
		Access
		Tourist infrastructure
3	Agricultural activities (A)	Permanent irrigation
		Land used in agricultural activities in the reception basin
4	Size of watershed (S)	N/A
5	Transportation infrastructure (T)	Railways
		Roads
6	Industrial activities (I)	Industrial activities
		Exploitation activities
7	Coverance with natural vegetation (C)	N/A

**Table 3:** Indicators used in the calculation of Hazard Index (HI).

No	Indicator
1	Land slope
2	Slope Aspect
3	Soil permeability

**Table 4:** Weights of indicators used in the calculation of the WRASTIC index.

Category of Use	Subcategory	Interval	Score	Weight
**Wastewaters** **(W)**	Aggregation nuclei	Natural Breaks (ArcGIS)	1–4	3
	Treatment plants	Primary processing	3	
		Secondary processing	2	
		Tertiary processing	1	
**Recreational activities** **(R)**	Aquatic sports	Motor-driven	5	3
		Non-motor-driven	4	
	Access	By car	3	
		Pedestrian	2	
		Prohibited	1	
	Tourist infrastructure	Present within 50 m	4	
		Absent within 50 m	0	
**Agricultural activities** **(A)**	Permanent irrigation	<10%	1	3
		10–25%	2	
		25–50%	3	
		50–75%	4	
		75–100%	5	
	Land used in agricultural activities in the reception basin	<20%	1	5
		20–40%	2	
		>30%	3	
**Size of watershed** **(S)**	N/A	<38.85 km^2^	1	1
		38.85 km^2^–155.39 km^2^	2	
		155.39 km^2^–388.47 km^2^	3	
		388.47 km^2^–1942.35 km^2^	4	
		>1942.35 km^2^	5	
**Ways of transport** **(T)**	Railways	Main railway line	4	1
		Tourist railway with narrow gauge	1	
	Roads	Highways or ring roads	5	
		National roads	4	
		County or local roads	3	
		Unpaved roads	1	
		No way of transport	0	
**Industrial activities** **(I)**	Industrial activities	Present	3	4
		Absent	0	
	Exploitation activities	Mines, quarries or landfills	5	
		Exploitation perimeters	1	
		No exploitation activity	0	
**Coverage with natural vegetation** **(C)**	N/A	<5%	5	1
		5–20%	4	
		20–35%	3	
		35–50%	2	
		>50%	1	

**Table 5:** Weights of indicators used in the calculation of Hazard Index.

Parameter	Interval	Score
Land slope	<the 25th percentile	1
	>the 25th, <the 50th percentile	3
	>the 50th, <the 75th percentile	4
	>the 75th percentile	5
Slope Aspect	Exposition privileges the accumulation of pollutants	5
	The exposition does not significantly affect the accumulation of pollutants	3
	The exposition does not privilege the accumulation of pollutants	1
Soil permeability	Clayish soil (smooth texture, low permeability)	5
	Sandy soil (sandy texture, average permeability)	3
	Gravel (rough texture, high permeability)	1

**Table 6:** The lake ecosystems evaluated for WRASTIC-HI.

No	Lake Name	Area (ha)	County	Origin	Included in Protected Areas	Morphologic Unit
1	Lake Voila	217	Brasov	Man made	Yes	Fagaras Depression
2	Lake Snagov	422	Ilfov	Natural	Yes	Snagov Plain
3	Lake Vacaresti	126	Dambovita	Man made	No	Targoviste Plain
4	Lake Vidraru	803	Arges	Man made	Yes	Lovistei Mountains
5	Lake Tau	78	Sibiu	Man made	Yes	Cindrel Mountains
6	Lake Firiza (Stramtori)	104	Maramures	Man made	No	Ignis Mountains
7	Lake Surduc	352	Timis	Man made	Yes	Lugojului Hills
8	Lake Taut	176	Arad	Man made	Yes	Tauti Depression
9	Lake Bezid	162	Mures	Man made	Yes	Tarnavelor Sub-Carpathian Region
10	Lake Lugasu	325	Bihor	Man made	Yes	Vad-Oradea Depression
11	Lake Stiucilor	31	Cluj	Natural	Yes	Sicului Hills
12	Lake Varsolt	324	Salaj	Man made	No	Simleu Depression
13	Lake Zanoaga Mare	6	Hunedoara	Natural	Yes	Retezat Mountains
14	Lake Oltina	1958	Constanta	Natural	Yes	Oltina Plateau
15	Lake Siutghiol	1756	Constanta	Natural	Yes	Istria Plateau
16	Lake Rosu	165	Harghita	Natural	Yes	Hasmas Mountains
17	Lake Lala	44	Bistrita-Nasaud	Natural	Yes	Rodna Mountains
18	Lake Bistret	409	Dolj	Natural	Yes	Bistretului Alluvial Plain
19	Lake Potcoava	90	Tulcea	Natural	Yes	Danube Delta
20	Lake Merhei	1385	Tulcea	Natural	Yes	Danube Delta
21	Lake Calimanesti	801	Galati	Man made	Yes	Siretului Plain
22	Lake Siriu	195	Buzau	Man made	Yes	Podu Calului Mountains
23	Lake Brates	2199	Galati	Man made	Yes	Brates Alluvial Plain
24	Lake Poiana Uzului	265	Bacau	Man made	No	Slanicului Hills
25	Lake Amara	700	Braila	Natural	Yes	Buzaului Alluvial Plain
26	Lake Razim	39,569	Tulcea	Natural	Yes	Danube Delta
27	Lake Solesti	374	Vaslui	Man made	No	Repedea-Zapodeni Plateau
28	Lake Bratul Dunarea Veche	186	Mehedinti	Natural	Yes	Drobeta-Bala Corridor
29	Lake Izvorul Muntelui	2843	Neamt	Man made	Yes	Ceahlau Mountains
30	Lake Stanca Costesti	4954	Botosani	Man made	Yes	Prut Corridor

**Table 7:** Results of the evaluation for WRASTIC index: Industrial activities (I); Recreational activities (R); Wastewater (W); Size of watershed (S); Ways of transport (T); Cover (vegetation) (C); Irrigation (a); Agricultural activities (A).

No	Lake Name	Results of the WRASTIC Index							
		(W)	(R)	(A)	(a)	(S)	(T)	(I)	(C)
1	Voila	0	5	3	2	1	1	0	2
2	Snagov	3	5	3	1	2	4	1	2
3	Vacaresti	3	5	5	2	1	3	3	4
4	Vidraru	2	5	1	1	2	4	0	1
5	Tau	2	5	1	1	1	4	0	1
6	Firiza	2	5	1	1	2	4	1	1
7	Surduc	3	5	3	2	2	3	0	1
8	Taut	3	5	3	2	2	3	0	1
9	Bezid	0	5	1	4	1	3	1	1
10	Lugasu	3	5	3	2	1	5	0	4
11	Stiucilor	2	5	5	3	1	4	0	3
12	Varsolt	3	5	5	1	1	4	1	2
13	Zanoaga Mare	0	2	1	1	1	1	0	2
14	Oltina	3	5	5	1	2	3	1	4
15	Siutghiol	2	5	3	1	2	5	3	4
16	Rosu	2	5	1	1	1	1	0	1
17	Lala	3	2	1	1	1	1	0	1
18	Bistret	3	2	1	1	1	4	1	5
19	Lake Potcoava	2	1	1	1	2	0	0	1
20	Lake Merhei	0	1	1	1	2	0	0	1
21	Calimanesti	2	5	5	1	2	3	3	2
22	Siriu	0	5	1	1	1	4	3	1
23	Brates	3	5	5	1	2	5	3	5
24	Poiana Uzului	2	5	1	1	2	3	1	1
25	Amara	3	5	5	1	2	4	0	4
26	Razim	0	5	1	1	4	4	0	4
27	Solesti	3	5	5	2	1	4	0	3
28	Bratul Dunarea Veche	1	1	1	2	1	4	5	1
29	Izvorul Muntelui	2	5	1	1	2	4	0	1
30	Stanca Costesti	0	5	3	1	1	0	0	4

**Table 8:** Results of the evaluation for HI index and degradation state.

No	Lake Name	Scores Obtained for HI			Degradation State
		Slope	Aspect	Permeability	
1	Voila	1	5	3	Semidegraded
2	Snagov	1	3	1	Semidegraded
3	Vacaresti	1	3	1	Degraded
4	Vidraru	4	5	1	Semidegraded
5	Tau	4	5	1	Semidegraded
6	Firiza	3	5	1	Semidegraded
7	Surduc	3	3	1	Semidegraded
8	Taut	3	3	1	Semidegraded
9	Bezid	3	5	1	Semidegraded
10	Lugasu	1	5	1	Semidegraded
11	Stiucilor	3	3	1	Semidegraded
12	Varsolt	1	5	1	Semidegraded
13	Zanoaga Mare	3	3	1	Natural
14	Oltina	3	5	1	Semidegraded
15	Siutghiol	2	3	1	Semidegraded
16	Rosu	4	5	1	Semidegraded
17	Lala	4	3	1	Natural
18	Bistret	1	3	5	Semidegraded
19	Lake Potcoava	1	3	1	Natural
20	Lake Merhei	1	3	1	Natural
21	Calimanesti	1	5	1	Semidegraded
22	Siriu	4	5	1	Semidegraded
23	Brates	1	5	5	Degraded
24	Poiana Uzului	3	5	5	Semidegraded
25	Amara	1	5	1	Semidegraded
26	Razim	1	3	1	Semidegraded
27	Solesti	3	5	1	Semidegraded
28	Bratul Dunarea Veche	1	5	1	Semidegraded
29	Izvorul Muntelui	3	3	1	Semidegraded
30	Stanca Costesti	1	3	1	Semidegraded

## Supplementary Materials

The following are available online at https://www.mdpi.com/article/10.3390/ijerph18115915/s1, Supplementary Table S1: TCW coefficients.
