# Supplementary material for: GIS-Based Multi-Criteria Analysis Method for Assessment of Lake Ecosystems Degradation—Case Study in Romania
Source: Int J Environ Res Public Health. 2021 May 31;18(11):5915. doi: 10.3390/ijerph18115915 (PMC8198268; doi:10.3390/ijerph18115915)
Supplement: Supplementary file 1 [file ijerph-18-05915-s001.zip › ijerph-1198632-supplementary.pdf]

Supplementary data

GIS based multi-criteria analysis method for assessment of lake ecosystems degradation - case study Romania

Sorin Avram [1,2], Corina Cipu [1], Ana-Maria Corpade [1,3], Carmen Adriana Gheorghe [1], Nicolae Manta [4], Mihaita-Iulian Niculae [1,5], Ionuţ Silviu Pascu [1,6,*], Róbert Eugen Szép[7,8,9] and Steliana Rodino [10,11,*]

[1] National Institute for Economic Research "Costin C. Kiriţescu" (INCE), Romanian Academy, 13 September Street, No. 13, 050711 Bucharest, Romania; avram.sorin@ucv.ro (S.A.); corina.cipu@upb.ro (C.C.); ana.corpade@ubbcluj.ro (A.-M.C.); carmen.adriana@ince.ro (C.A.G.);
[2] Department of Geography, University of Craiova, Al. I. Cuza Street, No. 13, 200585 Craiova, Romania
[3] Faculty of Geography, Babes-Bolyai University, Clinicilor Street, No 5-7, 400006 Cluj-Napoca, Romania
[4] Department of Biodiversity, Ministry of Environment, Waters and Forests, Libertăţii Boulevard, No. 12, 040129 Bucharest, Romania; nicumanta79@gmail.com
[5] Centre for Environmental Research and Impact Studies, University of Bucharest, Nicolae Balcescu Boulevard No. 1, 010041 Bucharest, Romania; mihaitaiulian.niculae@g.unibuc.ro (M.-I.N.)
[6] Department of Forest Monitoring, National Institute for Research and Development in Forestry "Marin Drăcea", 128 Eroilor Blvd., 077190 Voluntari, Ilfov, Romania
[7] Department of Bioengineering, Faculty of Economics, Socio - Human Science and Engineering, Sapientia Hungarian University of Transylvania, Piaţa Libertăţii 1, 530104, Miercurea Ciuc, Romania; szeprobert@uni.sapientia.ro
[8] Doctoral School of Chemistry, Faculty of Natural Sciences, University of Pécs, Ifjúság 6, 7624, Pécs, Hungary
[9] Institute for Research and Development for Hunting and Mountain Resources, Progresului 35B, 530240 Miercurea Ciuc, Romania.
[10] National Institute of Research and Development for Biological Sciences, Spl. Independentei, nr 296,
060031 Bucharest, Romania
[11] Institute of Research for Agriculture Economy and Rural Development, Bd. Marasti, nr 61, 011464 Bucharest, Romania
* Correspondence: ionut.silviu.pascu@gmail.com (I.S.P.); Steliana.rodino@yahoo.com (S.R.); Tel.: +40-726-332-258 (I.S.P.); +40-742-090-021 (S.R.)

The method used in this study for initial data structure design is derived from that proposed by Bangyu, 2016) [1]. The modular aspect, based on multiple indicators, takes into account the advantages of each method, canceling each other's disadvantages. Four stages were used to delimit the water bodies [2].

(i) Initially, a composite was made from the set of specific Landsat 8 strips, which would highlight the characteristics of the water against other classes. The use of bands 5, 6 and 4 as a substitute for the red, green and blue bands, which form an image in normal color, was the chosen version.

- Band 5 registers in the ideal spectrum of water identification, because it has a high degree of absorption in this interval;
- Band 6, is sensitive to turbidity and water content, while reducing the gap between urban and agricultural areas, facilitating subsequent separation into classes;

• Band 4, is sensitive to high chlorophyll content and environments with a high water content. Its disadvantage is the inability to differentiate the water vegetation, both being recorded in dark tones.

The next step was the unsupervised classification for an initial extraction of water bodies from the other classes. The method used was Iso Cluster, within ArcGIS. This unsupervised classification algorithm uses optimized iterations for grouping neighboring pixels based on the minimum Euclidean distance in a defined class and an unclassified pixel.

The result thus obtained was reclassified, thus eliminating the unfavorable classes and retaining the classes that define water. This new rasterial product was further used as a mask for a reclassification, this time supervised. In this case, the Maximum Likelihood method was used. The algorithm takes into account both the variance and the covariance of each class when associating a pixel. For this, it needs the spectral signatures of the pursued classes. For this purpose, the tools of the Image Classification tool in ArcGIS were used.

This stage aims to differentiate between water and clouds, respectively their shadow. The focus was on determining regions of interest in areas covered by water or shaded by clouds. Based on these, the spectral signatures used in the Maximum Likelihood algorithm were calculated. The result is transformed into a binary base, retaining values only for pixels that represent the presence of water. The function used is Raster to Float in Conversion.

(ii) The next phase was calculation of Normalized Difference Water Index (NDWI) by using the formula

$$(B3 - B5)/(B3 + B5) \qquad \text{Equation (1)}$$

Where B are Landsat 8 bands.

The result is reclassified, keeping only the values that define the water, which are then transformed as in the previous determination.

(iii) For the calculation of Tasseled Cap (TCW) a Python algorithm was developed. The analysis uses a linear combination between bands 2 - 7 of Landsat 8, using the formula

$$\text{Index} = (c2 * B2) + (c3 * B3) + (c4 * B4) + (c5 * B5) + (c6 + B6) + (c7 + B7) \qquad \text{Equation (2)}$$

Where c are coefficients for brightness, greenness, wetness and B are Landsat 8 bands [3]. The values of the coefficients are taken from the literature (Supplementary Table 1).

Supplementary Table 1 TCW coefficients

Index	Band 2	Band 3	Band 4	Band 5	Band 6	Band 7
Brightness	0,3029	0,2786	0,4733	0,5599	0,508	0,1872
Greenness	–0,2941	–0,243	–0,5424	0,7276	0,0713	–0,1608
Wetness	0,1511	0,1973	0,3283	0,3407	–0,7117	–0,4559

(iv) As a last step, a rasterial calculation was performed that took into account the advantages and disadvantages of the three methods used. Through the reunion (Mosaic to New Raster) between reclassified TCW and reclassified NDWI, all water bodies are obtained, clearly delimited, with the disadvantage of confusing them with shadows and clouds in the satellite image. However, subsequently applying an

intersection between the result and the binary image of the Maximum Likelihood classification, only those water bodies are extracted that overlap with the supervised determination that clearly identifies the boundary between water and shadow. Thus, a result is obtained characterized by the spatial resolution of the TCW, the sensitivity in the infrared spectrum (hence implicitly to humidity) of the NDWI and the supervised delimitation of the Maximum Likelihood.

The obtained raster was transformed into polygons via Raster to Polygon and was compared with the Copernicus database.

Thus, the equivalence in number of geometries between determinations and an even better spatial resolution of Copernicus products could be observed. This data set was validated for use in the GIS analysis stage of the degradation state of lake ecosystems.

References

1. Bangyu, L.; Zhang, H.; Fanjiang, X., 2. Water Extraction in High Resolution Remote Sensing Image Based on Hierarchical Spectrum and Shape Features, *Earth and Environmental Science* **2014**, 17.

2. Avram, S.; Croitoru, A.; Gheorghe, C., Manta, N. Cartarea ecosistemelor naturale și seminaturale degradate, Romanian Academy publishing House, 2018, p 132.

3. Baig, M.H.A.; Zhang, L.; Shuai T., Tong, Q Derivation of a tasselled cap transformation based on Landsat 8 at-satellite reflectance, *Remote Sensing Letters* **2014,** 5:5, 423-431, DOI: 10.1080/2150704X.2014.915434
